# *Edwardsiella tarda* outbreak affecting fishes and aquatic birds in Brazil

**DOI:** 10.1080/01652176.2018.1540070

**Published:** 2019-01-22

**Authors:** Yamê Miniero Davies, Maria Gabriela Xavier de Oliveira, Marcos Paulo Vieira Cunha, Leticia Soares Franco, Sandy Lorena Pulecio Santos, Luisa Zanolli Moreno, Vasco Túlio de Moura Gomes, Maria Inês Zanolli Sato, Marcello Schiavo Nardi, Andrea Micke Moreno, Andre Becker Saidenberg, Lilian Rose Marques de Sá, Terezinha Knöbl

**Affiliations:** a Departamento de Patologia, Faculdade de Medicina Veterinária e Zootecnia da, Universidade de São Paulo, São Paulo, Brazil;; b Departamento de Medicina Veterinária Preventiva e Saúde Animal, Faculdade de Medicina Veterinária e Zootecnia da, Universidade de São Paulo, São Paulo, Brazil;; c Companhia Ambiental do Estado de São Paulo (CETESB), São Paulo, Brazil

**Keywords:** *Edwardsiella*, edwardsiellosis, enterobacteria, fishes, birds, public health

## Abstract

**Background:***Edwardsiella tarda* infections are frequent causes of severe outbreaks in the fish farming industry besides representing possible zoonotic risks. However, naturally occurring outbreaks that affect various species besides fishes are seldom described.

**Aim:** To report an outbreak of acute mortality caused by *E. tarda* affecting multiple species that inhabited a natural pond in the state of São Paulo, Brazil.

**Materials and methods:** Three adult tilapias, three Mallard ducks and one Snow egret were necropsied and subjected to further microbiological tests. Gross and microscopic lesions were documented. The antibiotic susceptibility and phylogenetic similarities among fish and avian strains were also determined. The *E. tarda* species was confirmed through MALDI-TOF, partial *sodB* sequencing and phylogenetic analysis.

**Results:** Macroscopical findings between the three species included intestinal dilatation, mucosal hyperaemia and mucous to liquid contents. Common histopathology findings included acute enteritis, increased number of intraepithelial lymphocytes with bacteria adhered to the intestinal epithelium and lymphoid depletion in the spleen. *E. tarda* was isolated from several organs from all affected species. The phylogeny employing amplified fragment length polymorphism (AFLP) of eleven strains revealed high similarity (>90%) among the isolates regardless of the affected species or sampled organs. Ten isolates of *E. tarda* showed susceptibility to all tested antibiotics.

**Conclusions:***E. tarda* was identified as the cause of death of the species examined. Further studies would be necessary to determine the virulence of these strains and the possible risks regarding public health.

## 1. Introduction

*Edwardsiella tarda* are intracellular Gram-negative bacilli belonging to the Enterobacteriaceae family (Ewing et al. 1965). This bacterium is widely found in the freshwater and marine environment, possessing a wide geographical distribution. Although found as part of this environment and being isolated from asymptomatic animals, it may cause intestinal and extra-intestinal infections, acting as an opportunistic pathogen in immunocompromised or injured individuals both in the aquatic and terrestrial settings (Coles et al. 1978; Janda and Abbott 1993; Xu and Zhang 2014).

*E. tarda* is a frequent cause of severe economic losses to the fish farming industry around the world. It generally affects fishes maintained in inadequate aquatic environmental conditions with high temperatures and high concentration of organic material (Park et al. 2012). Clinical signs may vary according to age and species affected and externally fishes may present loss of skin pigmentation, abdominal distension, cutaneous haemorrhagic lesions and rectal prolapse. Common internal lesions in fishes include ascites, abscesses, peritonitis, hepatic, splenic and renal congestion (Miyazaki and Kaige 1985; Pavanelli et al. 1998).

Besides representing a great cause of concern to aquaculture, *E. tarda* occasionally causes infections in humans (Janda and Abbott 1993). The course of infection is dependent on the immunological status of the individual and linked to the exposure to the aquatic environment, contact with exotic animals such as fishes, amphibians or reptiles, or ingestion of raw fish meat (Janda and Abbott 1993; Novotny et al. 2004). The bacterium is suggested as an emerging gastrointestinal pathogen and the scale of fish farming tends to increase its zoonotic potential with this industry’s products serving as a reservoir for human outbreaks (Leung et al. 2012).

The agent can be detected in asymptomatic aquatic birds besides being also capable of causing disease in these animals. Clinical signs of emaciation, dyspnoea, diarrhoea, pale mucous membranes and sepsis are frequently reported, while necropsy findings generally include hepatomegaly and congestion of gastric and intestinal mucosa (Leotta et al. 2009; Campos et al. 2013).

Current studies referring to outbreaks of *E. tarda* in animals are mostly focused on a single studied species or aim to describe the carrier state by diverse species (Baya et al. 1997; Leotta et al. 2009; Xu and Zhang 2014). Nevertheless, edwardsiellosis outbreaks that may represent important epidemiological events and that affect multiple vertebrate species sharing the same aquatic environment are seldom reported (White et al. 1973). In this context, here we describe a mortality outbreak associated with *E. tarda* affecting fish as well as domestic ducks and a wild egret sharing a pond located on a subsistence farm in the state of São Paulo, Brazil. Gross and microscopic lesions were documented. The antibiotic susceptibility and phylogenetic similarities among fish and avian strains were also determined.

## 2. Materials and methods

Between March and May 2016, 30 tilapias, 10 Mallard ducks (*Anas platyrhynchos domesticus*) and one Snowy egret (*Egretta thula*) were reported to die on acute mortality event. These animals used the same pond on a farm that maintains diverse species which also visited the pond, and its immediate settings such as dogs, cats, swine, bovine, goats, sheep and horses. No treatment, management of water quality and waste drained to the pond was attempted previously.

Three adult individuals of tilapias, three Mallard ducks and one Snowy egret were necropsied and subjected to further microbiological tests at the Laboratory of Experimental and Compared Gastroenterology and Environmental Pathology and to the Avian Medicine Laboratory of the Pathology Department (School of Veterinary Medicine and Animal Science), University of São Paulo, Brazil.

Necropsy procedures evaluated the organ alterations according to size, colour, topography and consistency. Samples from all organs were fixed in 10% neutral buffer formalin and routinely processed for microscopical evaluation, stained with haematoxylin-eosin (HE).

Sterile swabs were taken from the trachea, air sacs, pericardium, cardiac blood as well as fragments of organs (liver, intestines, stomach, gills, spleen), and intestinal contents, conserved at 10 °C and sent to the Avian Medicine Laboratory. Cultures were performed by firstly incubating in brain and heart infusion broth (BHI, Oxoid™, Basingsktoke, UK) and isolation on MacConkey and 5% sheep blood agar, incubating at 37 °C for 24 h.

The selected colonies were identified to species level by matrix-assisted laser desorption-ionisation-time of flight (MALDI-TOF) mass spectrometry following the protein extraction according to described protocols (Kuhnert et al. 2012). The captured spectra were loaded in MALDI BioTyper™ version 3.0 software (Bruker Daltonik, Bremen, Germany) for bacterial identification.

For the *Edwardsiella* species confirmation, *sodB* sequencing was performed on all studied isolates. DNA extraction was performed according to Boom et al. (1990) protocol. Yamada and Wakabayashi (1999) primers were applied for partial gene amplification and sequencing was performed by the Human Genome Research Centre (University of São Paulo, Brazil). A phylogenetic tree was constructed using the maximum-likelihood method by Mega version 5.10 (https://www.megasoftware.net/home) (Tamura et al. 2011) and 500 bootstrap replicates were used for branch support statistical inference. The DNA sequences from this study were deposited in GenBank under accession numbers MH427728-MH427737.

The resistance profile to antibiotics was determined employing the disc-diffusion technique following the recommendations of the Clinical Laboratory Standards Institute (CLSI 2008). Impregnated antibiotic discs included enrofloxacin, ampicillin, gentamicin, nalidixic acid, cefotaxime, amoxicillin + clavulanic acid, streptomycin, co-trimoxazole and tetracycline.

*E. tarda* isolates were genotyped using the amplified fragment length polymorphism (AFLP) technique employing the restriction enzyme HindIII (New England BioLabs, Ipswich, MA) according to the protocol of Mclauchlin et al. (2000). The resulting fragments were analysed with the Bionumerics™ version 7.6 software (Applied Maths, Sint-Martens-Latem, Belgium) employing the Dice coefficient and the unweighted pair-group method using arithmetic average (UPGMA) to construct the dendrograms.

## 3. Results

**Figure 1.**
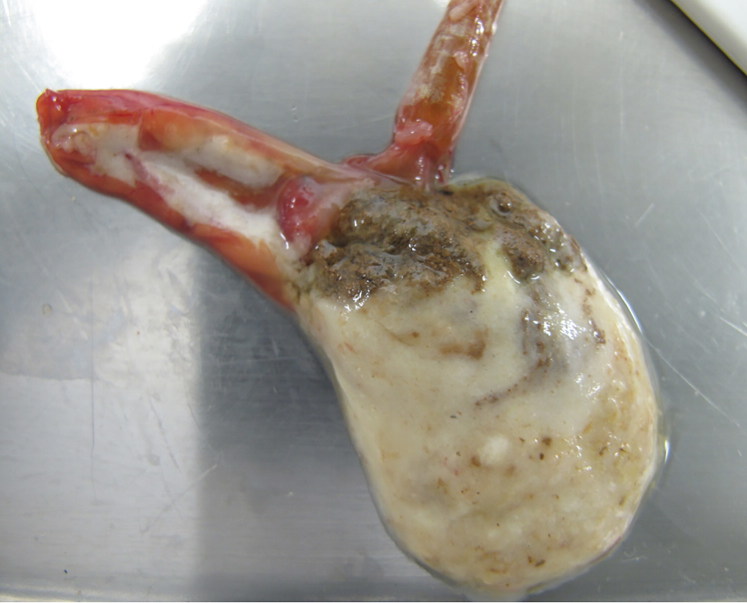
Tilapia. Gastric and intestine dilatation with abundant presence of mucous.

**Figure 2.**
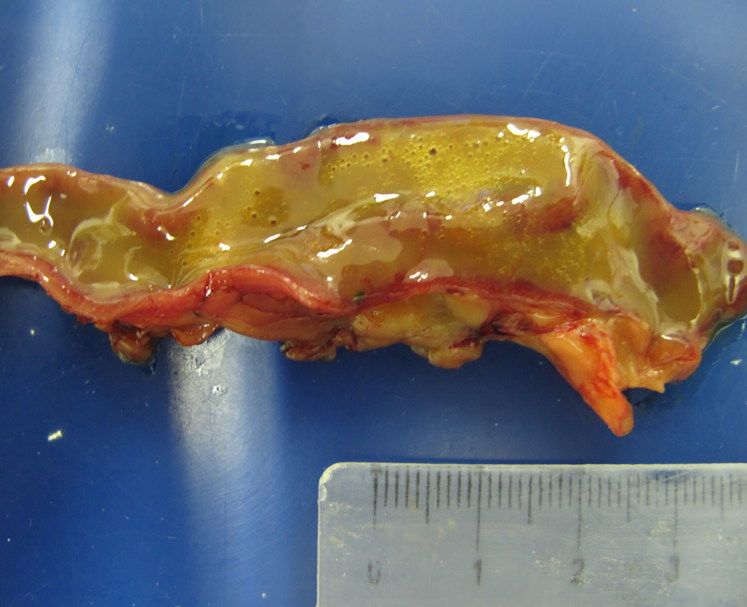
Duck. Hyperaemic intestinal mucosa and watery to mucous contents.

Common macroscopic results from birds and fishes alike showed good corporal condition, intestinal dilatation with a hyperaemic mucosa and filled with mucous to liquid contents. Tilapias showed gastric dilatation and abundant greyish mucous contents in the beginning of intestine (Figure 1). Two tilapias presented ascites with serosanguineous fluid. Affected Mallard ducks presented hyperaemic intestinal mucosa and mucous contents (Figure 2).

**Figure 3.**
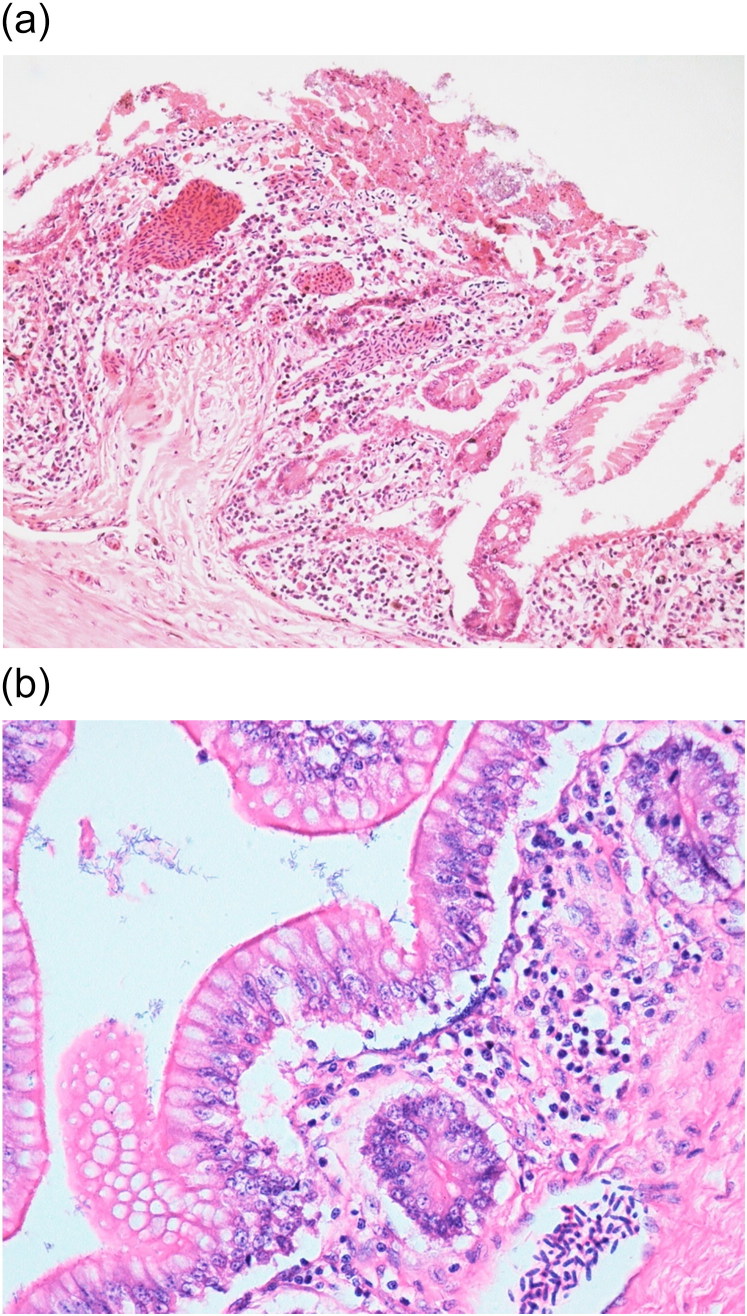
(a) Duck. Intestine. Lymphocytic enteritis with bacteria and cell debris in the intestinal lumen, diffuse marked necrosis in mucosa. H&E. 20X. (b) Duck. Intestine. Lymphocytic enteritis with bacteria adhered to intestinal mucosa. H&E. 40X.

**Figure 4.**
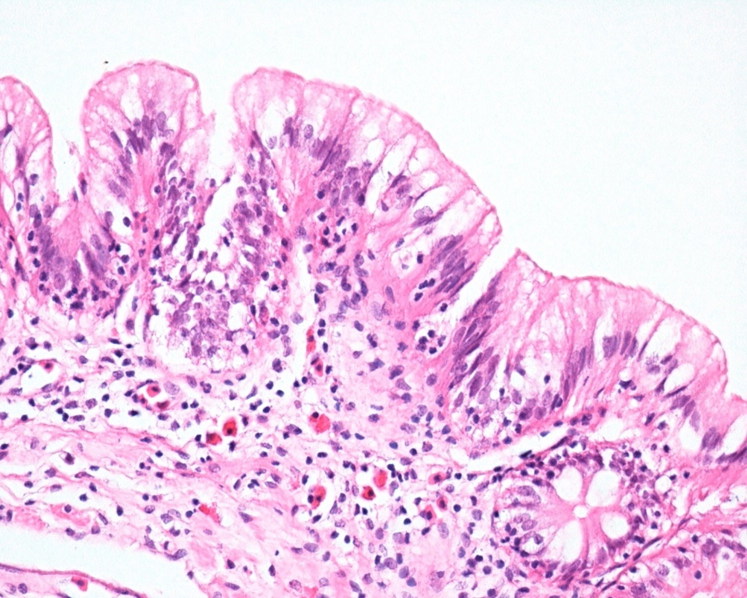
Tilapia. Intestine. Acute lymphocytic enteritis. Lymphocytic infiltrate in intestinal epithelial and lamina propria, mixed with granular leukocytes in lamina propria. H&E. 400X.

**Figure 5.**
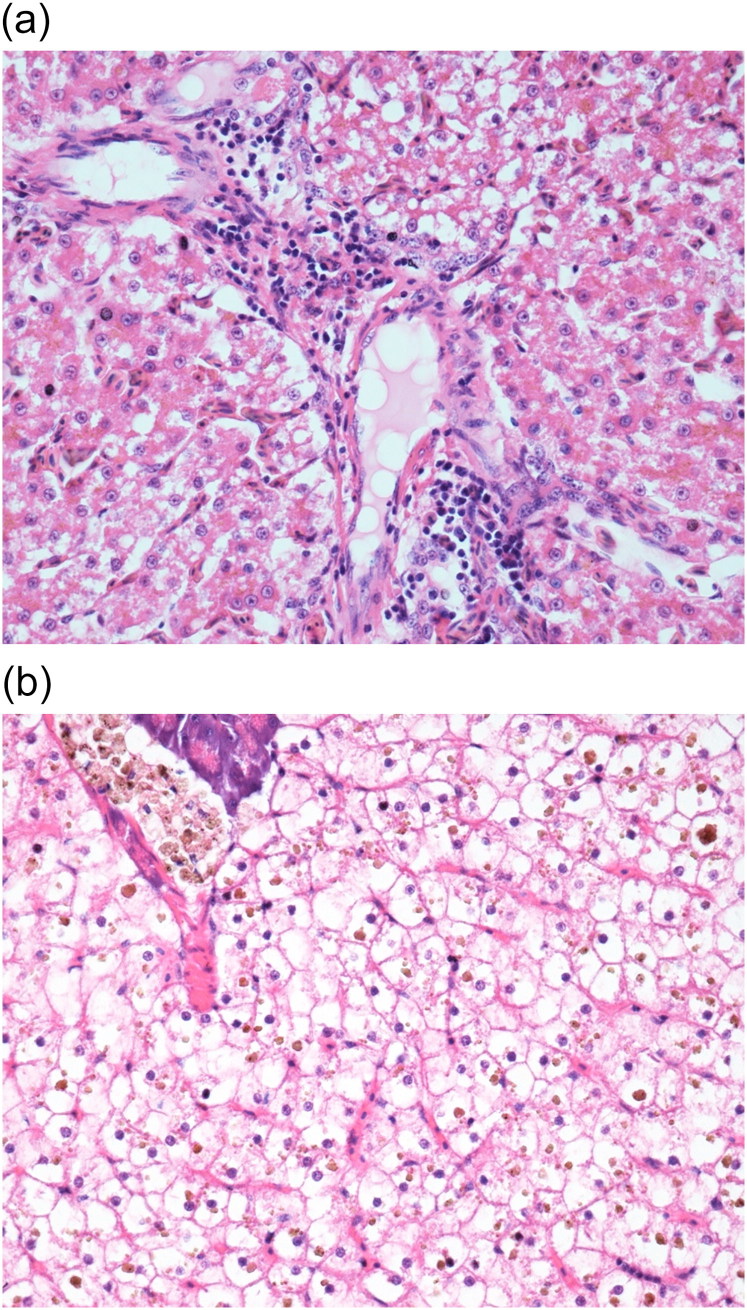
(a) Duck. Liver. Discrete lymphocytic portal hepatitis. H&E. 40X. (b) Tilapia. Hepatopancreas. Diffuse cellular swelling, presence of intrahepatocytic pigment deposits. Presence of melanomacrophage centres (MMC). H&E. 40X.

**Figure 6.**
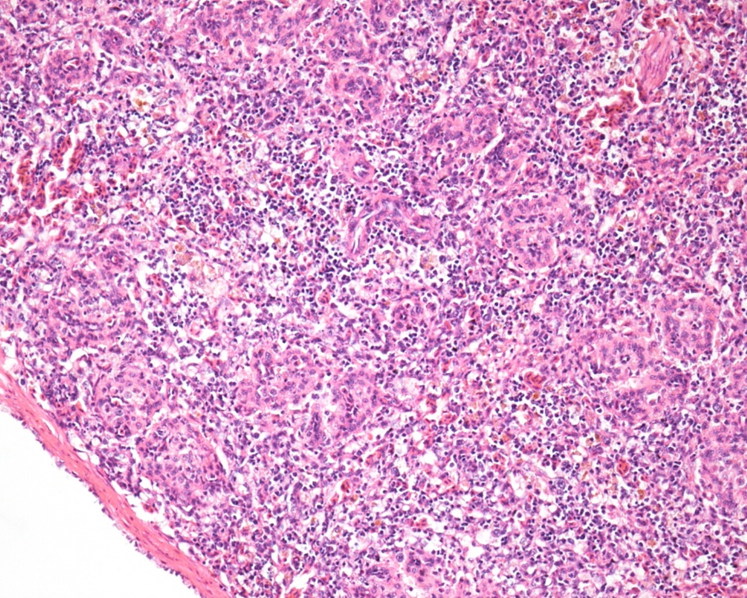
Egret. Spleen. Moderate diffuse lymphoid depletion. H&E. 20X.

**Figure 7.**
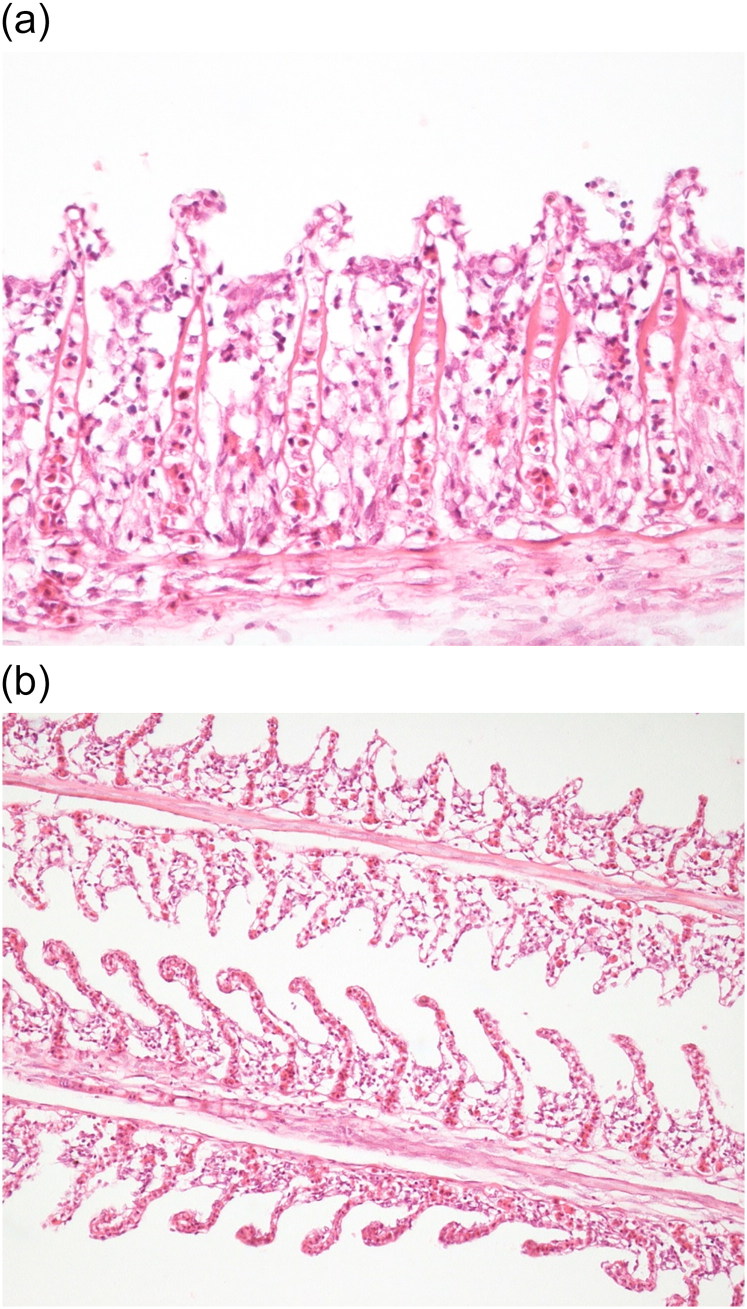
(a) Tilapia. Gill. Fusion and atrophy of secondary lamellae, epithelial hypertrophy. H&E. 40X. (b) Tilapia. Gill. Rupture of cells pillars in secondary lamellae, partial fusion of secondary lamellae. H&E. 20X.

Common major histopathological findings in aquatic birds and fishes were in intestine (6/7) characterised by acute enteritis. In aquatic birds (2 ducks and 1 egret), there was increased number of heterophils, lymphocytes and plasma cells in lamina propria associated with congested mucosa and multifocally necrotic hypereosinophilic epithelial cells sloughed into the intestinal lumina. There was multiple focus of bacteria adhered to the intestinal epithelium (Figure 3(a,b)). In tilapias (3/3), diffuse acute enteritis (Figure 4) was characterised by increased number of lymphocytes in lamina propria and among epithelial cells in intestinal mucosa; focus of granular leukocytes in lamina propria, bacterial adhered to the intestinal epithelium and focus of sloughed necrotic epithelial cells into the intestinal lumina. Other histopathological findings in the aquatic birds were portal lymphoid hepatitis (Figure 5(a)), haemorrhage in kidneys, lungs and brain and lymphoid depletion in the spleen (Figure 6). In tilapias, the main lesions detected in other organs were: diffuse cellular swelling of hepatocytes, steatosis and presence of melanomacrophage aggregates (Figure 5(b)), haemorrhage in kidneys, lymphoid depletion and haemorrhage in spleen and rupture of pillar cells, lamellar epithelial hypertrophy and fusion of secondary lamellae in the gills (Figure 7(a,b)).

**Figure 8.**
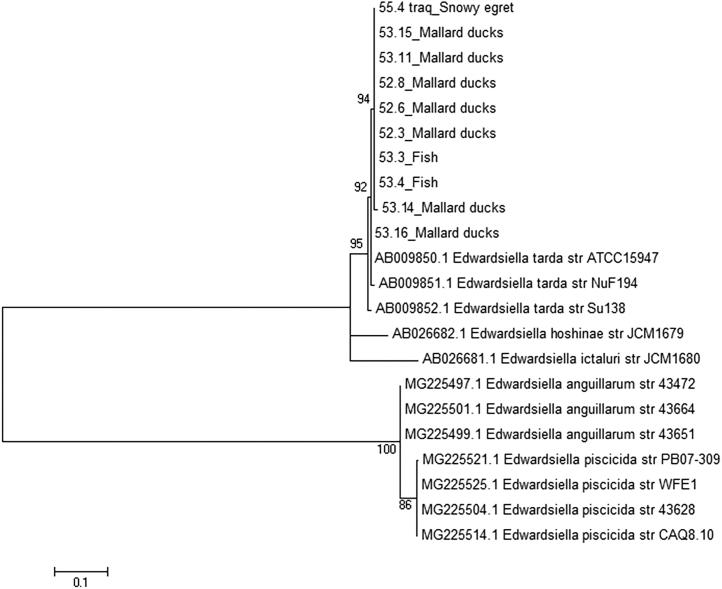
Amplified fragment length polymorphism profiles (AFLP) obtained with *E. tarda* strains.

**Figure 9.**
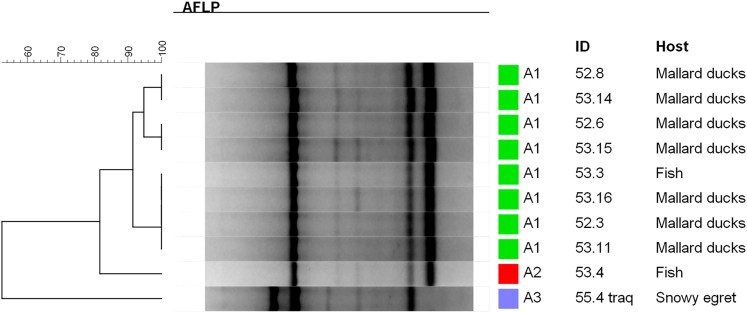
*sodB* phylogeny demonstrating species confirmation for studied *E. tarda* strains. The bootstrap values are presented at the corresponding branches.

The bacterial cultures and subsequent identification of species by MALDI-TOF revealed the presence of diverse Enterobacteriaceae among the sampled organs. *E. tarda* was identified in several organs from all species (Table 1; Figure 8). The *E. tarda* species was confirmed through partial *sodB* sequencing and phylogenetic analysis (Figure 9). Specifically, the agent was isolated from the gills, stomach, intestines and spleen from the tilapias; and from the cloaca, pericardium and intestines from ducks. The tracheal and cloacal samples of the egret were also positive for *E. tarda*.

**Table 1.** Bacterial species found on each sampled species and respective sampled site.

Organ	Tilapias	Mallard ducks	Snowy egret
Gills	*Escherichia coli* Edwarsiella tarda*Plesiomonas shigelloides	–	–
Stomach	*Escherichia coli* Edwarsiella tarda* *Bacillus cereus*	–	–
Intestine and cloaca	*Proteus vulgaris* *Morganella morganii* Edwarsiella tarda* *Escherichia coli*	*Escherichia coli* Edwarsiella tarda* *Enterococcus faecalis* *Enterococcus faecium*	*Escherichia coli* Edwardsiella tarda* *Enterococcus faecalis*
Trachea	–	*Aeromonas hydrophila* *Klebsiella pneumoniae* *Escherichia colli* *Morganella morganii*	*Citrobacter freundii* *Serratia marcescens* *Klebsiella pneumoniae* Edwardsiella tarda*
Spleen	Edwarsiella tarda*	–	–
Pericardium	–	Edwarsiella tarda* *Acinetobacter bawmanni* *Escherichia coli* *Enterobacter aerogenes* *Enterococcus hirae*	–

*positive for Edwardsiella tarda.

Ten isolates of *E. tarda* showed susceptibility to all tested antibiotics. Phylogenetic comparison using AFLP exhibited high degree of similarity (>90%) among strains, regardless of the species or organ where they were isolated (Figure 8).

## 4. Discussion

Although *E. tarda* is well studied due to its importance as a fish pathogen and in human clinical cases, naturally occurring outbreaks that simultaneously affect fishes, domestic ducks and wild birds are not frequent in literature. Here, *E. tarda* was considered the cause of acute septicaemia in tilapias, ducks and egret. Even though other *Enterobacteriaceae* with pathogenic potential were isolated, *E. tarda* was the only pathogenic microorganism isolated from all organs presenting morphological changes as described.

Gross results from deceased fishes revealed ascites and gastrointestinal alterations which partly include the characteristic edwardsiellosis lesions that have been previously reported in diverse fish species (Muratori et al. 2001; Park et al. 2012), including tilapias (Pavanelli et al. 1998). The main reported lesions were linked to the virulence of specific strains, affected fish species and the acute course of infection as other authors had hypothesised (Garcia et al. 2012). Studies involving different strains of *E. tarda* have identified specific virulence factors that facilitate the colonisation and invasion which result in diverse clinical manifestations and lesions (Leung et al. 2012; Xu and Zhang 2014).

Common microscopic lesions reported in fishes infected by *E. tarda* mostly include suppurative interstitial nephritis, hepatitis and purulent splenitis (Mohanty and Sahoo 2007; Park et al. 2012). However, here tilapias showed significant alterations in the intestinal tract which was the major organ affected by the infection. Muratori et al. (2001) reported an edwardsiellosis outbreak in Brazil, affecting farmed tilapia (*Oreochromis* spp.) in which hallmark lesions were intestinal lesions, including extensive haemorrhage. Therefore, it is interesting to notice how likely different strains may cause varied symptoms and lesions worldwide, as it was observed in the avian species analysed. It has been also suggested that the similarity in the distribution and associated lesions of *E. tarda* reported between humans and tilapia could possibly justify the implementation of this species as an experimental animal model for the study of pathogenesis and therapeutic strategies in the control of edwardsiellosis (Garcia et al. 2012).

In this study, *E. tarda* was associated with acute enteritis in aquatic bird species including adult ducks and an egret. Mortality events affecting free-ranging adult aquatic birds have also been previously associated with *E. tarda*. Predisposing factors were difficult to establish in the outbreak. The water quality may have influenced the course of the outbreak due to high organic material concentration and high temperatures, which facilitate an exponential growth of *E. tarda* (White et al. 1973). These environmental conditions are also highlighted as contributing factors to common fish outbreaks and the bacterium is known to remain latent in a carrier state in fishes (Mohanty and Sahoo 2007; Park et al. 2012). In this report, the pond used by avian species had no specific water management or quality control and it received organic material waste from domestic animals from the farm, creating favourable conditions to high concentration of *E. tarda*.

Gross and histopathology results of ducks and egret agree to some extent with other authors that describing edwardsiellosis outbreaks of adult birds. These studies report a septicaemic process in most cases coupled with the fact that the intestines are particularly affected. Haemorrhagic intestinal lesions associated with the isolation of *E. tarda* were found in mortality events of free-ranging Brown pelicans (*Pelecannus occidentalis carolinensis*) (White et al.^ 1973^). While the necropsy of a stranded and weakened Magellanic penguin (*Spheniscus magellanicus*) received in a rehabilitation centre presented mainly gastric and intestinal lesions (Campos et al. 2013).

Although these reports illustrate *E. tarda* as a cause of infection in avian species, it has been shown that certain wild birds can also be asymptomatic carriers for this organism (Leotta et al. 2009). Therefore, a concomitant factor should be present to predispose avian species to develop an infection, which seems connected to a compromised or non-fully developed immune system (Campos et al. 2013). The conditions causing the acute infection in the current report could be likely related to the high concentration of the organism in the pond perhaps coupled with some unidentified environmental stressor.

The antibiotic susceptibility profiles observed for all strains are in agreement with other authors who describe *Edwardsiella* generally susceptible to tetracyclines, aminoglycosides, most beta-lactams and fluoroquinolones (Stock and Wiedemann 2001). However, there have been recent reports of drug-resistant isolates in fishes due to the scale and continuous growth of fish farming practices and the use of antibiotics to control these infections. This is accompanied by increased concerns of transmission of genetic resistance elements to other animal pathogens (including those affecting humans) as well as the resulting drug waste residues in the environment (Leung et al. 2012; Xu and Zhang 2014).

Our AFLP results showed >90% similarity for 8/10 isolates revealing a clonal spread between the diseased species. Interestingly, another study phylogenetically compared *E. tarda* from fish and human samples collected from both asymptomatic individuals and outbreaks using random amplified polymorphic DNA (RAPD). It was shown that the unrelated strains from diverse disease events and geographical origins were scattered but showed clusters of almost exclusive human or fish origins, thus segregating the lineages that caused infection on each species (Nucci et al. 2002). As opposed to that, our results revealed a clonal spread among fish and avian isolates showing that the origin for the outbreak was indeed the same and that our strains have the capacity to cross the fish/avian species barrier. Still, the pathogenic potential to mammals and therefore the zoonotic ability remains to be further studied.

## 5. Conclusion

Fishes, ducks and egret died due to gastrointestinal lesions and septicaemia as previously described for *E. tarda* infection, which was confirmed by bacterial cultures and species identification. The likely cause for the outbreak was an increase in numbers of *E. tarda* present in the pond due to the poor water quality. Further studies defining the virulence and their importance to public health would be important to better characterise these strains.
